# Synchronization by Food Access Modifies the Daily Variations in Expression and Activity of Liver GABA Transaminase

**DOI:** 10.1155/2014/590581

**Published:** 2014-04-07

**Authors:** Dalia De Ita-Pérez, Isabel Méndez, Olivia Vázquez-Martínez, Mónica Villalobos-Leal, Mauricio Díaz-Muñoz

**Affiliations:** Instituto de Neurobiología, Campus UNAM-Juriquilla, 76230 Querétaro, QRO, Mexico

## Abstract

Daytime restricted feeding (DRF) is an experimental protocol that influences the circadian timing system and underlies the expression of a biological clock known as the food entrained oscillator (FEO). Liver is the organ that reacts most rapidly to food restriction by adjusting the functional relationship between the molecular circadian clock and the metabolic networks. *γ*-Aminobutyric acid (GABA) is a signaling molecule in the liver, and able to modulate the cell cycle and apoptosis. This study was aimed at characterizing the expression and activity of the mostly mitochondrial enzyme GABA transaminase (GABA-T) during DRF/FEO expression. We found that DRF promotes a sustained increase of GABA-T in the liver homogenate and mitochondrial fraction throughout the entire day-night cycle. The higher amount of GABA-T promoted by DRF was not associated to changes in GABA-T mRNA or GABA-T activity. The GABA-T activity in the mitochondrial fraction even tended to decrease during the light period. We concluded that DRF influences the daily variations of GABA-T mRNA levels, stability, and catalytic activity of GABA-T. These data suggest that the liver GABAergic system responds to a metabolic challenge such as DRF and the concomitant appearance of the FEO.

## 1. Introduction

GABA transaminase (GABA-T; 4-aminobutanoate:2-oxoglutarate aminotransferase, EC 2.6.1.19) is a catabolic enzyme that converts GABA and *α*-ketoglutarate into succinate semialdehyde and glutamate. Its specificity is not strict since it can also recognize alanine, *β*-alanine, aspartate, and propanoate as substrates. GABA-T is dependent on pyridoxal-phosphate (PLP) as cofactor, and its catalytic mechanism consists of two coupled half reactions in which the PLP cofactor oscillates between the pyridoxal and the pyridoxamine forms [1]. GABA-T has been found in a large variety of species ranging from microorganisms, plants, invertebrates, and vertebrates. In eukaryotic cells, GABA-T is present, but not exclusively, within the mitochondria [2, 3]. This enzyme has been crystallized from several sources. The structure of GABA-T from brain pig indicates a *α*2 dimer with 472 residues per subunit; the two monomers are tightly intertwined, and the two PLP cofactors are located close to the subunit interface [4]. The structure of the brain GABA-T also contains a [2Fe-2S] cluster in the vicinity of the PLP cofactors [5]. The maturation process in the synthesis of GABA-T involves a proteolytic cleavage that removes the XRX(∗)XS motif. However, in the liver, GABA-T is further cleaved to a smaller isoform by a second proteolytic step catalyzed by a mitochondrial processing peptidase [6].

GABA is a well-known inhibitory transmitter in the nervous system. However, components of the GABAergic system have also been characterized in a variety of endocrine tissues such as pancreas, testis, and liver [7–9]. Mammalian liver contains GABA at nM levels as well as its specific transporters (GAT2) and receptors (both GABAR-A and GABAR-B) [10]. Liver GABA has been proposed to function as a positive regulator of the cell cycle, with implications in some forms of hepatocellular carcinoma [11]. High GABA-T activity has been reported in hepatic tissue [12], the rationale being that liver GABA-T mainly functions to degrade the GABA produced by microorganisms in the gastrointestinal tract [13]. In addition, GABA can also be present in the food ingested at mealtime.

Daytime restricted feeding (DRF) is an experimental protocol that influences the circadian timing system by promoting the emergence of an alternate master circadian oscillator that is independent of the suprachiasmatic nucleus (SCN) [14]. The basis for this new form of measuring the “physiological timing” is a circadian entity known as the food entrainable oscillator (FEO), but its anatomical location is still unidentified [15]. The DRF protocol involves the establishment of a catabolic response promoted by limiting the food intake to a few hours (2–4 h) during the light phase of the day-night cycle. The display of an anticipatory behavior is evident before mealtime (food anticipatory activity, FAA) as is a diversity of physiological and metabolic adaptations associated with DRF/FEO expression [16]. During the DRF protocol, some biochemical and structural changes in the liver have been associated with improved mitochondrial function as well as increased apoptotic activity and cell division, resulting in enhanced cellular exchange [17, 18].

To obtain further insights into the modulation of the mitochondrial adaptations of the liver GABA-handling system during the DRF protocol/FEO expression, we analyzed the daily variations of GABA-T mRNA and protein expression and enzymatic activity. The results evidenced under the DRF protocol are as follows: (1) an increased presence of GABA-T in homogenate and mitochondrial fraction, (2) changes in the rhythmic profile of GABA-T activity, and (3) disappearance of an ultradian rhythm of GABA-T mRNA.

## 2. Materials and Methods

### 2.1. Animals and Housing

Experiments were carried out with male Wistar rats weighing from 200 to 250 g and maintained under a 12:12 h LD cycle (lights on 08:00 h) at constant temperature (22 ± 1°C). Rats were kept in groups of 4 in transparent acrylic cages (40 × 50 × 20 cm) with free access to Purina Chow and water except during food restriction or fasting conditions. Experimental procedures were conducted in accordance with our Institutional Guide for Care and Use of Experimental Animals (Universidad Nacional Autónoma de México) and with international ethical standards [19].

### 2.2. Experimental Design

The experimental protocol has been published previously [20]. Briefly, rats were randomly assigned to one of the following feeding conditions for 3 weeks:

1. control animals fed with* ad libitum* with free access to food and water throughout the 24 h period, AL group;
2. experimental group with restricted food access (exclusively from 12:00 to 14:00 h), DRF group.

At the end of the 3 weeks, subgroups of animals were sacrificed at 3 h intervals over a complete 24 h cycle, starting at 08:00 h. The protocol was followed until the last day.

In addition, 2 more groups were included to compare the acute fasting and subsequent refeeding response in the DRF group.

1. Animals fed* ad libitum* were maintained with free food access for 3 weeks; on the last day, food was removed at 14:00 h, and, after 21 h (~1 day) of food deprivation, they were sacrificed (at 11:00 h), F group.
2. A second group of rats was similarly deprived of food for 21 h, then refed for 2 h (from 12:00 to 14:00), and sacrificed at 14:00 h, F + R group.

### 2.3. Liver Sampling and Subcellular Fractionation

Rats were beheaded with a guillotine-like instrument. The liver was removed (*≈*5 g) and immediately placed in an ice-cold isolation medium (1 : 10 wt/vol) containing 250 mM sucrose, 0.1% BSA (fatty acid free), and 0.5 mM EGTA (pH 7.4). The tissue was homogenized in 10 volumes of 10 mM Tris-HCl, pH 7.4, with a Teflon homogenizer (40 rpm for 10 s). Subcellular fractionation was done according to Aguilar-Delfín et al. [21]. Briefly, the homogenate was centrifuged at 1,500 g for 15 min, and the supernatant was centrifuged at 10,000 g for 20 min to sediment the mitochondrial fraction. Protein was measured by the method of Bradford [22].

### 2.4. GABA-T Activity

GABA-T activity was measured by a coupled enzymatic assay according to the method reported by Jung et al., 1977 [23]. The method involves the conversion of GABA to succinic acid by the consecutive reactions of GABA-T (in the sample) and semialdehyde dehydrogenase (added in the assay). As part of the reactions, NAD^+^ is reduced to NADH, allowing the quantification of GABA transamination by spectrophotometric recording at 340 nm. The increasing in optical density was recorded during the first 2 min of the assay.

### 2.5. Western Blot Analysis

Liver homogenates and mitochondrial fractions were obtained in RIPA buffer (Sigma-Aldrich, SLM, USA) and subjected to denaturing SDS-PAGE under reducing conditions. Total protein concentrations were determined by the Bradford method and equal amounts (30 *μ*g) were separated on 10% SDS-PAGE, transferred to nitrocellulose membranes, and blocked for 1 h in TBST buffer (20 mM TRIS, pH 7.5; 500 mM NaCl; 0.5% Tween 20) containing 5% nonfat milk. Membranes were then washed and incubated in the presence of mouse anti-GABA-T antibody (Rb mAb to ABAT, ab108249, Abcam, Cambridge, UK) diluted 1/10,000 in TBST overnight at 4°C. As controls, in the case of homogenates, membranes were incubated in the presence of mouse anti-tubulin antibody (ab56676, Abcam, Cambridge, UK) diluted 1/1,000 or in the case of mitochondrial fractions in the presence of rabbit anti-VDAC1/Porin antibody (ab15895, Abcam, Cambridge, UK) diluted 1/1,000. After washing, membranes were incubated with secondary antibodies conjugated to alkaline phosphatase (1/5,000). Bands were revealed using AP conjugate substrate kit (Bio-Rad, CA, USA). Densitometric analysis was performed using the Image Lab Software (v 3.0, Bio-Rad, CA, USA).

### 2.6. RT-qPCR Amplifications

GABA-T gene expression was evaluated by isolating total RNA from liver tissues (20–30 mg) using the SV Total RNA Isolation System (Promega, WI, USA). The amount and quality of RNA were estimated spectrophotometrically at 260 and 280 nm, and a constant amount of RNA (2 *μ*g) was reverse transcribed using SuperScript III Reverse Transcriptase, Oligo (dT) 12–18 Primer, RNaseOUT Recombinant Ribonuclease Inhibitor, and dNTP Set PCR Grade (Invitrogen, CA, USA). Amplification was performed in triplicate in the CFX96TM Real-Time PCR Detection System (Bio-Rad, CA, USA). Primers used for qPCR amplifications were synthesized by Sigma-Aldrich Co. (MO, USA), and the corresponding sequences were, for GABA-T (GenBank BC081787.1), forward 5′-TTCCGGAAGCTGAGAGACAT-3′ and reverse 5′-AGTCTGAACCTCGTCCACCA-3′ and, for ribosomal protein S18 (Rps18) (GenBank BC126072.1) used as housekeeping gene, forward 5′-TTCAGCACATCCTGCGAGTA-3′ and reverse 5′-TTGGTGAGGTCAATGTCTGC-3′. Amplifications were carried out with Maxima SYBR Green qPCR Master Mix (Thermo Fisher Scientific, MA USA) in a 10 *μ*L final reaction volume containing cDNA (1/100) and 0.5 *μ*M of each of the primer pairs in SYBR Green Master Mix, according to the following protocol: activation of Taq DNA polymerase and DNA denaturation at 95°C for 10 min, followed by 40 amplification cycles consisting of 10 s at 95°C, 30 s at 62°C, and 30 s at 72°C. The PCR data were analyzed by the 2^−ΔΔCT^ method and cycle thresholds (CT) normalized to the housekeeping gene Rps18 were used to calculate the mRNA levels of GABA-T.

### 2.7. Data Analysis

Data were classified by group and time and are displayed as mean ± standard error of the mean (SEM). Data were compared using a two-way ANOVA using Fisher's least significant difference (LSD) test for multiple and independent measures, with a factor for group (2 levels) and a factor for time (8 levels). In order to determine significant time effects for each daily sampling profile, a one-way ANOVA was performed for individual groups. The one-way ANOVA was followed by Tukey's post hoc test whereas the two-way ANOVA was followed by a Bonferroni post hoc test with the significance threshold set at *P* < 0.05 for both. The Student's* t*-test was applied to detect significant differences between DRF rats and the controls of feeding conditions (acute fasting and refeeding) both before and after food access (11:00 h and 14:00 h, resp.). The Student's* t*-test was also used to identify significant differences (*P* < 0.05) between the 24 h average values of the AL and DRF groups. Rhythmic analysis was performed by Chronos-Fit (v 1.06 developed by P. Zuther, S. Gorbey, and B. Lemmer, 2009) based on a partial Fourier analysis of the data [24]. All statistical analysis was performed with the program STATISTICA, version 4.5 (StatSoft Inc.).

## 3. Results

All results are double plotted displayed to have a better notion of the rhythmic profiles.

### 3.1. Liver GABA-T Is Increased by DRF

**Figure 1:**
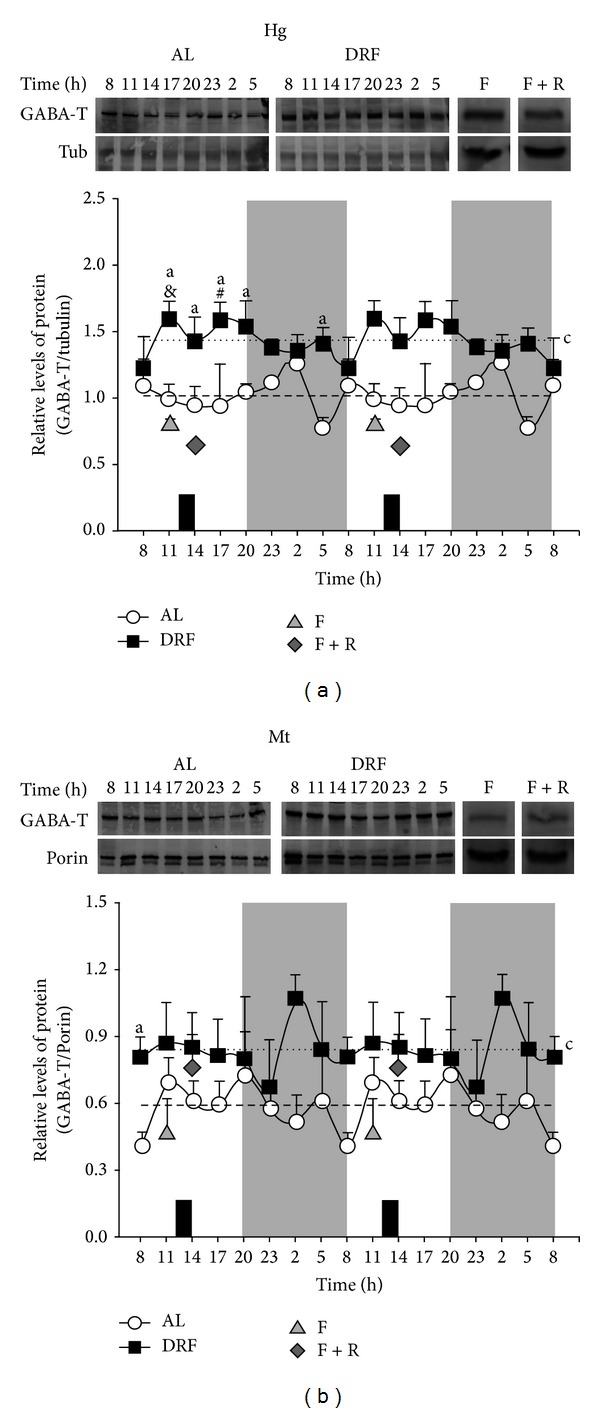
Western blot analysis of 24 h profile of GABA-T protein in liver of AL and DRF rats. Liver homogenates (Hg) (a) and mitochondrial fractions (Mt) (b) were subjected to electrophoresis on 10% SDS-polyacrylamide gels. Data were quantified by densitometry of the bands obtained from homogenates (a) and mitochondrial fractions (b) collected over a 24 h period from the AL and DRF groups and food condition controls, F and F + R. Food availability for the DRF group is indicated by dark boxes (from 12:00 to 14:00 h). Average values are represented as a dashed line for the AL group and as a dotted line for the DRF group. Graphs represent the mean ± SEM of 4 rats per time point. Significant differences (*P* < 0.05) are indicated as follows: a: DRF versus AL by two-way ANOVA and post hoc Bonferroni test; c: mean of DRF versus mean of AL by Student's* t*-test for both homogenate and mitochondrial fraction; for homogenates only: &, DRF versus F at 11:00; #, DRF versus F + R at 14:00, both by the Student's* t*-test.

The amount of GABA-T protein detected by Western blot analysis in the liver homogenate and mitochondrial fraction is shown in Figure 1. GABA-T did not show any rhythmicity in liver homogenate from AL rats (a). DRF rats showed a significant increase in GABA-T protein throughout the 24 h cycle. The average value was ~38% higher in the DRF group than in the AL group (Table 1 and Figure 1(a)). In addition, significant differences (higher levels in DRF than AL rats) were detected at 05:00 h, 11:00 h, 14:00 h, 17:00 h, and 20:00 h. Similar to the AL rats, no rhythmicity was detected in the Western blot signal in the homogenate of the DRF group. The level of GABA-T was responsive to acute 24 h fasting (F group), showing a significant reduction in comparison to the DRF group at 11:00 h. Two hours of feeding after fasting (F + R group) were not enough to restore the low levels of GABA-T associated with the acute fasting condition (a).

Since GABA-T is in an important proportion a mitochondrial enzyme, the amount of this protein was also evaluated by Western blot analysis in the liver mitochondrial fraction in the AL and DRF groups (b). As observed in liver homogenate, GABA-T was significantly increased in the DRF group. However, comparing the calculated average values, the elevation was more moderate (~26%, Table 1). Again, no rhythmicity was detected in either group. A significant difference between both groups was detected at 02:00 h. In contrast to the effect observed in the liver homogenate, neither acute fasting (group F) nor acute fasting followed by 2 h of refeeding (F + R group) modified the amount of GABA-T protein in the mitochondrial fraction. However, as to the controls of acute feeding conditions, there was a significant increase (~85%) in the F + R rats in comparison to the F rats.

### 3.2. The Activity of Liver GABA-T in the Mitochondrial Fraction Was Modified by DRF

**Figure 2:**
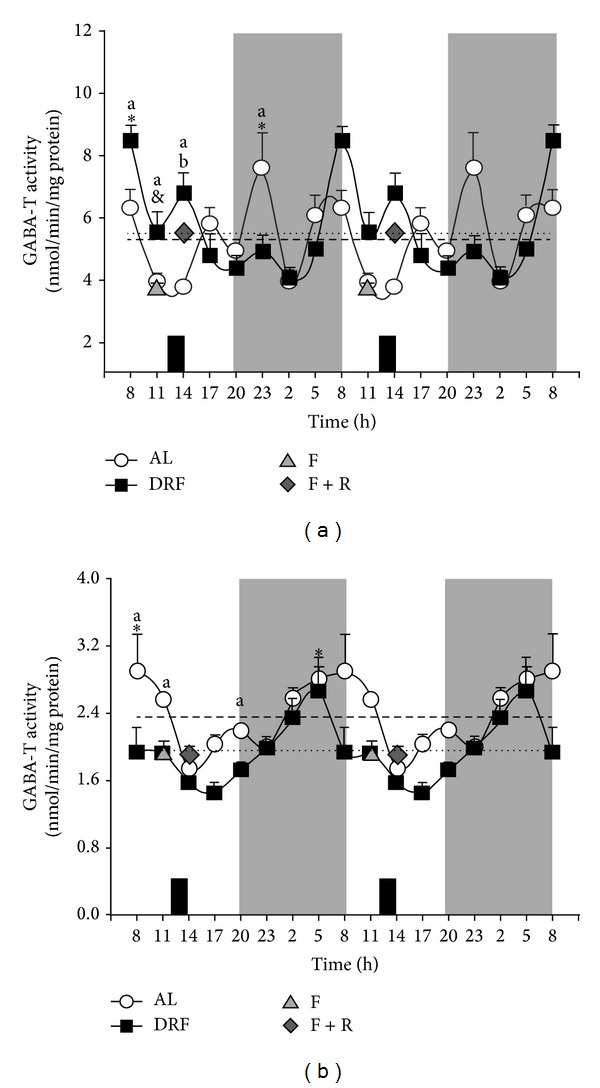
Analysis of 24 h profile of GABA-T activity in the liver of AL and DRF rats. GABA-T activity was measured in both the homogenate (a) and mitochondrial fraction (b) of the livers from AL and DRF rats by a spectrophotometric assay over a 24 h period. Rats were subjected to a 12 h : 12 h regime of light : dark. The shaded zone represents the dark phase. Food availability for RFS group is indicated by dark boxes (from 12:00 to 14:00 h). Controls of food condition are shown (F and F + R). Average values are represented as dashed line for AL group and a dotted line for RFS group. Graphs show the mean ± SEM of 4 rats per time point. Significant differences (*P* < 0.05) are indicated as follows: ∗: DRF versus AL by one-way ANOVA and post hoc Bonferroni test, in both the homogenate and mitochondrial fraction; a: RFS versus AL by two-way ANOVA and post hoc Bonferroni test in the mitochondria fraction only; b: F versus F + R by Student's* t*-test.

The activity of GABA-T in AL and DRF groups is shown in Figure 2 for the liver homogenate (a) and for the mitochondrial fraction (b). GABA-T activity in the homogenate fluctuated in both groups, with peaks at 23:00 h and 08:00 h in AL and DRF rats, respectively (Table 1). At these times, significant differences were detected between both groups. In contrast, GABA-T activity in the mitochondrial fraction tended to be reduced in the DRF group, and both groups showed rhythmicity. Peak value for AL rats was at 08:00 h and for DRF rats was at 05:00 h (Table 1). A clear sinusoidal pattern was observed in the AL group with the lowest value in the middle of the light phase and the peak at the transition from the dark and to the light period. The DRF group also exhibited a sinusoidal pattern with the lowest value near the end of the light period and the peak 12 h later. However, this pattern was consistent with the lower values of mitochondrial GABA-T activity, especially during the light period.

In both liver homogenate and the mitochondrial fraction, the food condition controls (F and F + R groups) did not show any changes in comparison to the AL and DRF rats at 11:00 h and 14:00 h, respectively.

### 3.3. mRNA of Liver GABA-T Was Not Rhythmic in the DRF Group

**Figure 3:**
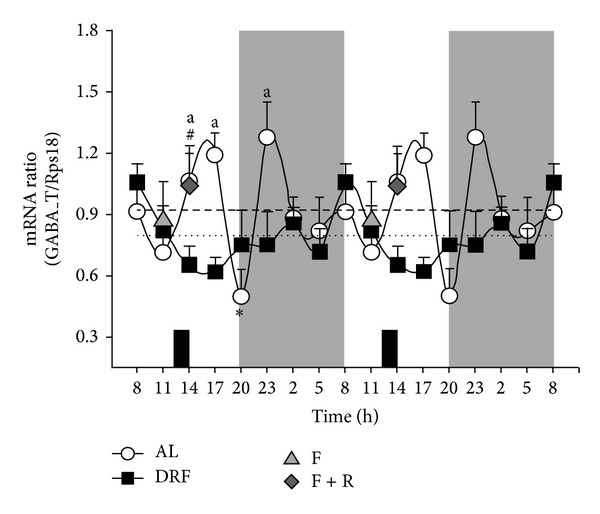
Analysis of the 24 h profile of relative mRNA expression of GABA-T in the liver of rats under AL and DRF conditions. Relative mRNA levels were determined by RT-qPCR and normalized to Rps18 expression. The shaded zone represents the dark phase. Food availability for DRF group is indicated by dark boxes (from 12:00 to 14:00 h). Graphs show the mean ± SEM of 6 to 8 rats per time point. Average values are represented as a dashed line for the AL group and a dotted line for DRF group. Significant differences (*P* < 0.05) are indicated as follows: ∗: DRF versus AL by one-way ANOVA and post hoc Bonferroni test, in both the homogenate and mitochondrial fraction; a: RFS versus AL by two-way ANOVA and post hoc Bonferroni test; #: DRF versus F + R at 14:00, by the Student's* t*-test.

The mRNA for GABA-T was quantified in liver by RT-qPCR in the AL and DRF groups, and the results are shown in Figure 3. AL rats showed rhythmicity in this parameter with 2 larger peaks, one at 17:00 h and the second at 23:00 h, and a minor peak at 08:00 h. There was also a pronounce valley at 20:00 h. Indeed, rhythmic analysis detected an ultradian rhythm with a period of ~8 h (Table 1). DRF rats did not show any rhythmicity. Significant differences between AL and DRF groups were detected at 14:00 h, 17:00 h, and 23:00 h, being higher the values from the AL rats. No significant differences in the expression of GABA-T mRNA were detected between the F and DRF group at 11:00 h as well as between the F + R and DRF group at 14:00 h.

## 4. Discussion

### 4.1. Liver GABAergic System

Our data show that under a protocol of daytime restricted feeding (DRF) with the subsequent appearance of the feeding entrained oscillator (FEO) the properties of liver GABA-T are affected and highlight the plasticity of the hepatic GABAergic system under a metabolic challenge.

It is well documented that GABA and its metabolizing enzymes are found outside the nervous system and play a role in the cell signaling of several organs and tissues [25]. These tissues express the anabolic and catabolic enzymes for GABA, and measurable levels of this molecule can be detected [7, 12]. It has also been reported that hepatic tissue expresses elements of the GABAergic system, such as specific receptors (GABAR-A and GABAR-B), transporters, and the catabolic enzyme GABA-T [10]. Hepatocytes and other liver cell types are clearly in contact with GABA, which may be produced by the liver itself or may come from the microbial production of the intestinal tract [13, 25]. In this context, it has been suggested that GABA present in hepatic tissue could act as a regulator of the cell cycle and the migration of cancerous hepatocytes [11, 26]. As a signaling molecule, liver GABA must be finely controlled by plasma membrane transporters but, mainly, by its catabolic transformation by GABA-T [27].

### 4.2. Liver GABA Transaminase

GABA-T is, but not exclusively, a mitochondrial enzyme that requires PLP for its activity. In the liver, it exists as a smaller variant because of proteolytic processing that removes 12–14 amino acids [6]. The pharmacological inhibition of GABA-T has been explored for several years, motivated by attempts to find an effective antiepileptic drug [28]; however, not much information has been reported regarding its transcriptional regulation or the modulation of its activity by covalent modifications.

Our results indicate that liver GABA-T activity, mainly in the mitochondrial fraction, and the level of GABA-T mRNA in AL groups show daily fluctuations. Previously, our group reported that the mitochondrial yield after cellular fractionation did not change by the DRF protocol [17]. The data also show that the DRF protocol affected the properties of hepatic GABA-T at different levels. (1) The elevated levels of this protein enzyme in the liver homogenate and mitochondrial fraction (Figure 1) did not correlate with the corresponding levels of mRNA (Figure 3). A possible explanation is that the restricted feeding schedule promoted an increased stability of GABA-T or an increment in its translational rate. (2) The higher amount in the presence of GABA-T did not correlate with the GABA-T activity measured in liver homogenate and the mitochondrial fraction (Figure 2). This lack of correspondence could be explained by the existence of a negative regulator of GABA-T activity, such as a covalent modification or some other type of posttranslational processing. We have not yet found any reports of phosphorylation, acetylation, methylation, or other modulatory modifications of GABA-T activity. However, these modifications in GABA-T properties promoted by the protocol of DRF/FEO expression can be considered as a metabolic adaptation based on the biochemical plasticity of this enzymatic system.

### 4.3. GABA-T under Food Synchronization/Food Entrainable Oscillator

The changes in the amount and activity of GABA-T associated with the protocol of daytime restricted feeding are part of an extensive set of metabolic and physiological adaptations that occur in the liver and other organs when the daily rhythmicity is modified by a new organization of the timing system [29, 30]. The synchronization imposed by offering food for only 2–4 h per day is a powerful timing cue that, in some circumstances, overrules the control of the master pacemaker, the SCN [31]. This situation elicits food anticipatory activity as well as the expression of a biological clock (the FEO) that is independent of the SCN. The anatomical localization of the FEO is unknown, but the hypothesis guiding our research is that it is an emergent oscillator distributed among hypothalamic/midbrain nuclei that control food intake and peripheral organs such as liver, adrenals, stomach, and others important for nutrient processing [15, 32].

It is in the liver where the influence of the FEO is most evident. The liver plays central roles in both the processing of nutrients arriving from the duodenal tract and the modulating of the hunger-satiety cycle [33]. This fact is especially relevant during DRF because of the marked hyperphagia after the 2 h of food access. Indeed, the intense arrival of nutrients during the restricted feeding could be a factor in the daily entrainment showed by the liver [34].

In previous reports, we have shown that DRF/FEO expression strongly modifies the metabolic status of the liver mitochondria, promoting a more oxidized state and increasing the synthesis of ATP [17, 35]. In addition, DRF/FEO expression is associated with an elevation of the malate-aspartate shuttle activity [17]. Together these data indicate that liver mitochondria are responsive to the adaptations associated with FEO expression, as an example of the plasticity shown by the timing system during the metabolic adaptations associated with restricted feeding schedules. This is of particular interest to the present project since GABA-T is mostly a mitochondrial enzyme.

### 4.4. Rheostatic Adaptation

The exact molecular mechanisms that underlie the adaptive modifications of liver physiology during expression of the FEO and in response to the DRF protocol are not known. However, an initial rationale towards the identification of this sequence of events is to postulate that synchronizing circadian physiology by limited food access has an impact on the reciprocal regulation between energetic metabolic networks and the cellular timing system [36, 37]. During DRF/FEO expression, the connection between the molecular circadian clock and the metabolic activities has been thought to adopt a new relationship that is different from that in the control condition of* ad libitum* feeding. This new interaction has the characteristics of an emergent property, since it is not observed in the* ad libitum* condition or in the control groups of acute fasting and acute fasting followed by refeeding. For example, the increased amount GABA-T protein promoted by the protocol of DRF/FEO expression and detected by Western blot analysis (Figure 1) is unique and is observed neither in the AL group nor in the acute control. The same stands for the augmented GABA-T activity in liver homogenate and the loss of rhythmicity in GABA-T mRNA. Again, all these changes can be considered as part of the plastic adaptations that are present in the liver during the expression of the FEO.

One way to consider the emergence of new properties in biological systems is the concept of rheostasis [38], which is a term used in the theory of physiological control to describe regulation around shifting set points. In contrast to homeostasis, rheostasis triggers associations in situations with potentially adjustable settings [32]. In this context, expression of the FEO could involve a novel rheostatic adaptation in conditions in which two contradictory environmental temporal clues, light-dark cycle and DRF, are coexisting [39].

## 5. Conclusions

Biochemical properties of liver GABA-T were modified by the DRF protocol. An explanation for these findings should consider the coincidence of the FEO expression with a functional light-driven SCN. In addition, GABA-T regulation could be responding to the intense arrival of nutrients during the 2 h of food access, in the context of restricted feeding schedules. The data raise the possibility that alterations in the hepatic GABAergic system could be among the metabolic and physiological adaptions that occur in the liver during expression of the FEO. This possibility needs further exploration.

## Figures and Tables

**Table 1:** Chronobiological analysis of liver GABA-T parameters: mRNA, protein amount, and activity.

	GABA-T mRNA		GABA-T protein				GABA-T activity			
			Hg		Mt		Hg		Mt	
	AL	DRF	AL	DRF	AL	DRF	AL	DRF	AL	DRF
Rhythm (%)	—	—	—	—	—	—	—	54.8	40.3	38.4
Mesor	0.9	0.8	1.1	1.5	0.7	0.9	5.2	5.4	2.4	2.0
Amplitude	0.3	—	—	—	—	—	—	1.55	0.48	0.50
Acrophase (h:min)	7:40/15:15/22:50	—	—	—	—	—	—	9:56	5:56	4:14
Period	8 h	—	—	—	—	—	—	24 h	24 h	24 h

Chronos-Fit analysis was performed to evaluate rhythmicity for GABA-T parameters in liver. Acrophases in GABA-T mRNA from AL group were repeated each 7 h with 40 min. Hg: liver homogenate, Mt: mitochondrial fraction, AL: ad libitum, and DRF: daytime restricted feeding. (—) means that no rhythmic pattern was detected.
